# 

**Published:** 1899-09

**Authors:** 


					SEPTK1BEK, 1899.
THE
AMERICAN m -M-h 1-
?w- O F -w-j
DENTAL SGIENCEt
EDITED BY
F. J. S. GORGAS, M. D., D. D. S.
RICHARD GRADY, M. D., D. D. S.
Vol. 33.
THIRD SERIES
No. 5.
BALTIMORE!
SNOWDEN 4. COWMAN MFG. CO., 9 W. Fayette St.
LONDON'.
TRUBNER &. CO., 60 Paternoster Row.
Entered at the Post Office at Baltimore, Md., as Second-class Matter.
PRYRBLE IN ^DV^-NCE.
IPUBLISHED MONTHLY
IS2.50 PER HNNUM.

				

